# Supplementary material for: Formic Acid Decomposition Using Palladium-Zinc Preformed Colloidal Nanoparticles Supported on Carbon Nanofibre in Batch and Continuous Flow Reactors: Experimental and Computational Fluid Dynamics Modelling Studies
Source: Nanomaterials (Basel). 2023 Nov 22;13(23):2993. doi: 10.3390/nano13232993 (PMC10708417; doi:10.3390/nano13232993)
Supplement: Supplementary file 1 [file nanomaterials-13-02993-s001.zip › nanomaterials-2658668-supplementary.pdf]

Supplementary Materials

Formic Acid Decomposition Using Palladium-Zinc Preformed Colloidal Nanoparticles Supported on Carbon Nanofibre in Batch and Continuous Flow Reactors: Experimental and Computational Fluid Dynamics Modelling Studies

Sanaa Hafeez [1], Eleana Harkou [2], Panayiota Adamou [2], Ilaria Barlocco [3], Elisa Zanella [3], George Manos [4], Sultan M. Al-Salem [5], Xiaowei Chen [6], Juan Josè Delgado [6], Nikolaos Dimitratos [7,8], Alberto Villa [3] and Achilleas Constantinou [2,*]

[1] School of Engineering and Materials Science, Queen Mary University of London, London E1 4NS, UK; sanaa.hafeez@qmul.ac.uk
[2] Department of Chemical Engineering, Cyprus University of Technology, 57 Corner of Athinon and Anexartisias, Limassol 3036, Cyprus; ea.harkou@edu.cut.ac.cy (E.H.); paa.adamou@edu.cut.ac.cy (P.A.)
[3] Department of Chemistry, University of Milan, Via Golgi, 20133 Milan, Italy; ilaria.barlocco@unimib.it (I.B.); elisa.zanella@unimi.it (E.Z.); alberto.villa@unimi.it (A.V.)
[4] Department of Chemical Engineering, University College London, London WCIE 7JE, UK; g.manos@ucl.ac.uk
[5] Environment & Life Sciences Research Centre, Kuwait Institute for Scientific Research, P.O. Box 24885, Safat 13109, Kuwait; ssalem@kisr.edu.kw
[6] Department of Materials Science Metallurgical Engineering and Inorganic Chemistry, University of Cádiz, Campus Río San Pedro, E-11510 Puerto Real, Spain; xiaowei.chen@uca.es (X.C.); juanjose.delgado@uca.es (J.J.D.)
[7] Department of Industrial Chemistry "Toso Montanari", Alma Mater Studiorum University of Bologna, Viale Risorgimento 4, 40136 Bologna, Italy; nikolaos.dimitratos@unibo.it
[8] Center for Chemical Catalysis-C3, Alma Mater Studiorum University of Bologna, Viale Risorgimento 4, 40136 Bologna, Italy
* Correspondence: a.konstantinou@cut.ac.cy

Contents

1. Materials and Methods
   - 1.1 Materials
   - 1.2 Catalyst preparation
   - 1.3 Catalyst characterisation
   - 1.4 Catalytic tests
     - 1.4.1 Batch reactor
     - 1.4.2 Packed bed reactor
   - 1.5 Product analysis
2. Modelling methodology
   - 2.1 Reaction pathway
   - 2.2 Batch reactor modelling
   - 2.3 Packed bed reactor modelling
3. Results
   - 3.1 Catalyst characterisation
   - 3.2 Packed bed flow reactor validation

Supplementary figures

Figure S1. Schematic diagram of the batch reactor set-up.

Figure S2. Schematic diagram of packed bed reactor set up.

Figure S3. XPS analysis of Pd_6Zn_4 catalyst.

Figure S4. Comparison between the single and double membrane configurations on the formic acid conversion at different temperatures [8].

1. Materials and Methods

1.1 Materials

Zinc Chloride ($ZnCl_2$, 98%), potassium tetrachloropalladate (II) (K_2PdCl_4, 99.99%) and sodium borohydride ($NaBH_4$, 99.99%) were used without any pre-treatment for the catalysts synthesis and they were purchased from Sigma-Aldrich (Haverhill, MA, USA). All the catalytic tests were carried out using FA (≥95%, Sigma-Aldrich) and deionised water as the substrate and the solvent, respectively. CNFs PR24-HHT (High Heat-Treated carbon nanofiber) were obtained from Applied Science Company (Cedarville, OH, USA).

1.2 Catalyst preparation

The following procedure was employed to synthesise 0.5 g of 2 wt% bimetallic catalysts Pd6Zn4 supported on HHT-CNFs. The whole process was conducted in inert atmosphere in order to avoid Zn oxidation. 500 mL of milliQ water were previously degassed in vacuum for 1h and then purged in N2 for 30 minutes. At this point, 100 mL of degassed milliQ water were used to previously disperse 0.5 g of support, due to the hydrophobic behaviour of HHT. The solution of the two precursor salts (6.67 x 10-2 mmol of Pd and 4.44 x 10-2 mmol of Zn) were added into a baker containing the remaining 400 mL of milliQ water. Then, polyvinyl alcohol (M/PVA = 1/0.65) was added. After 3 minutes NaBH4 (metal/NaBH4 1/8 mol/mol) was added to the mixture and the instantaneous formation of metallic colloid was proved by the change in colour of the solution, which became darker. After 30 minutes, , the support was added to the collodail solution in order to anchor the NPs. Sulfuric acid (H2SO4) was added to the suspension until pH 2 and stirred for 30 minutes to guarantee the total immobilisation of NPs. The solid was then filtered, washed with 0.5 L of deionised water and dried in oven at 80 °C for 48 h obtaining the final catalytic materials.

1.3 Catalyst characterisation

X-ray photoelectron spectroscopy (XPS) measurements were performed using a Thermo Scientific K-alpha+ spectrometer equipped with a monochromatic Al X-Ray source operating at 72 W, with the signal averaged over an oval-shape area of 600x400 μ. Data were recorded at 150 eV for survey scans and 40 eV for high resolution (HR) scans with a 1 eV and 0.1 eV step size, respectively. CASAXPS (v2.3.17 PR1.1) was used for the analysis of the data. Transmission electron microscopy (TEM) experiments were performed on a double Cs aberration-corrected FEI Titan3 Themis 60–300 microscope equipped with a mono-chromator, a X-FEG gun and a high efficiency XEDS ChemiSTEM, which consists of a 4-windowless SDD detectors. HR-STEM imaging was performed at 200 kV and using a high-angle annular dark-field (HAADF) detector with a camera length of 11.5 cm. XEDS mappings were performed using a beam current of 200 pA and a dwell time per pixel of 128 μs. To im-prove the visual quality of the elemental maps obtained, these were filtered using a Gaussian blur of 0.8 using Velox software. Based on the STEM-HAADF images of the catalysts, the diameters of more than 200 metal particles randomly selected were measured and the corresponding metal particle size distributions (PSD) were determined. Based on these PSDs, the average particle diameter (d) was calculated according to the following expression: d = ∑nidi/∑ni, where ni ≥ 200. Likewise, the total metal dispersion was calculated according to D = Ns/Nt, where Ns is total number of surface metal atoms and Nt is total number of atoms in the metal particle. For particle size calculation ImageJ software was used.

1.4 Catalytic tests

1.4.1 Batch reactor

FA transformation was carried out in a two-necked round bottom flask containing a reflux condenser and a magnetic stirrer and placed in a water bath. 10 mL of an aqueous solution of FA was added in the reactor. Different concentrations of the FA solution were utilised, 0.5, 0.4, 0.3, 0.2 and 0.1 M. Upon reaching the chosen temperature, the catalyst with a FA:metal molar ratio of 2000:1 was added and the mixture was stirred at 1400 rpm. The effect of the reaction temperature was investigated, varying from 30 to 60°C, where the temperature within the reactor was measured using

thermocouple and was found to be uniform. The reaction conditions have been selected based on optimization from previous reported papers from our group [1]. A schematic diagram of the batch reactor set-up is depicted in Figure S1.

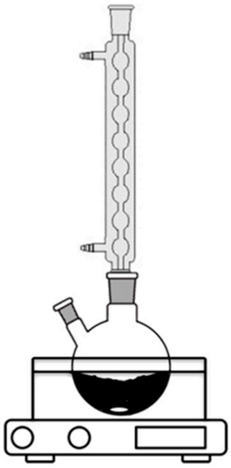

Figure S1. Schematic diagram of the batch reactor set-up.

1.4.2 Packed bed reactor

Liquid-phase FA dehydrogenation in a fixed bed reactor was tested with a bed length of 7 cm (50 mg 2%Pd_6Zn_4/HHT-CNFs and 50 mg HHT-CNFs). The bed was heated to a constant reaction temperature of 30 °C. 0.1 M FA was inserted into the reactor with a flow of 0.1, 0.3 and 0.5 mL min^{-1}. Figure S2 presents the schematic diagram of packed bed reactor set-up.

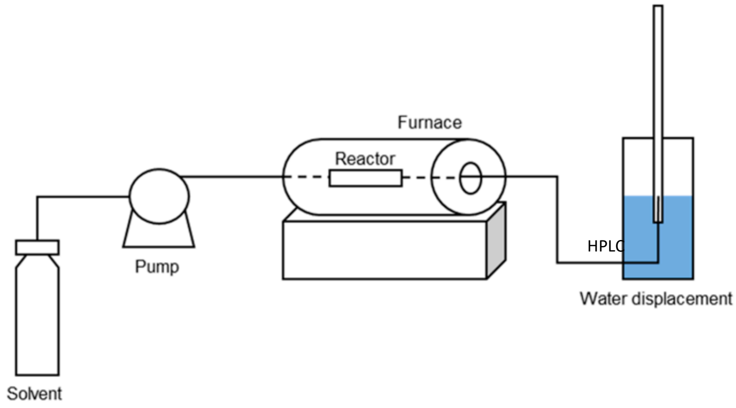

Figure S2. Schematic diagram of packed bed reactor set up.

1.5 Product analysis

An H+ chromatographic column was employed (Alltech OA- 10,308, 300 mm 7.8 mm) with UV detector settled at 210 nm. Liquid samples of 200 μL were withdrawn periodically and diluted to 5 mL with the eluent of the analysis, a H_2SO_4 solution (0.005 N). An online mi-cro-gas chromatograph (Agilent 3000A) equipped with two different columns: (a) a molecular sieve module and (b) an OV-1 module (stationary phase of polydimethylsiloxilane), and a TCD detector, was used to analyse the gas phase (CO_2 and CO) after 2h of reaction. Measure of CO_2 and CO were performed using calibration curves obtained from commercial standards.

2. Modelling methodology

2.1 Reaction pathway

The decomposition of FA follows two pathways, depending on factors such as the catalytic material, reaction temperature, and the reactants concentrations. Equation (1) represents the dehydrogenation of FA, while Equation (2) illustrates the dehydration reaction. Since the first reaction is slightly exothermic and the second reaction is endothermic, the choice of catalyst can influence which pathway is more likely to occur in the decomposition of FA[2], [3].

$$HCOOH \rightarrow CO_2 + H_2 \tag{1}$$

$$HCOOH \rightarrow CO + H_2O \tag{2}$$

In a previous experimental study [4], the results of the decomposition of FA were fitted to the power-law model formula, therefore the reaction rate presented below was used for the model.

$$r = k \times C^n \tag{3}$$

where r is the reaction rate, k is the reaction rate constant, C is the FA concentration and n is the order of reaction. The order of the reaction was calculated by the previous study and the estimated value is 0.2, same with the Pd/C catalyst, which validated the experimental results accurately. The activation energy was calculated by the experimental data obtained from this study, approximately at 22 kJ/mol.

2.2 Batch reactor modelling

In the case of the batch reactor, a closed, ideally mixed system of constant volume is assumed, since no flow enters or leaves the system. Reactants in gas phase are assumed to be ideal gases while reactants in liquid phase assumed to be incompressible and ideal. Based on these assumptions; the decomposition of FA can be modelled in 0D adopting the Batch reactor feature using the Reaction Engineering interface of the Chemical Reaction Engineering Module in COMSOL.

The mass balance equation for each species, *i*, that is solved in the Reaction Engineering interface is expressed as:

$$\frac{dc_i}{dt} = R_i \tag{4}$$

where c_i is the components molar concentration (mol/m^3) and R_i represents the sum of each reaction's contribution to the reaction rate (mol/(m^{3}s)). By default, the energy balance for the batch reactor is calculated according to:

$$V_r \sum_i c_j C_{p,i} \frac{dT}{dt} = Q + Q_{ext} + V_r \frac{dp}{dt} \tag{5}$$

where V_r is the reactor volume (m^3), $C_{p,i}$ denotes the molar heat capacity of the components (J/(mol·K)), T is the temperature (K), and p the pressure (Pa). On the other side of the formula, Q is the heat due to chemical reaction (J/s), and Q_{ext} signifies the external heat (J/s). The heat of reaction can be expressed as shown in the equation below:

$$Q = -V_r \sum_i H_i r_i \tag{6}$$

where H_i is the molar enthalpy (J/mol), and r_i is the reaction rate (mol/(m^{3}s)).

2.3 Packed bed reactor modelling

A 2D configuration was used to model the packed bed microreactor assuming that temperature, mass, and velocity profiles occur only in the radial and axial directions. Further hypotheses were made for the modelling of the packed bed microreactor model such as (a) unsteady-state and laminar flow; (b) isothermal operation of the microreactor; (c) fluids in the gas phase behave as ideal gases; (d) constant axial fluid velocity with uniform physical properties and transport coefficients. The solid catalyst utilised for the study is Pd_6Zn_4@HHT (spherical particles), and deionised water is used as a solvent.

The mass balance equation given below incorporates the diffusion and convection for the species in the packed bed:

$$\frac{\partial c_i}{\partial t} + \nabla \cdot J_i S_b + u \cdot \nabla c_i = R \quad (7)$$

where u is the velocity (m/s) and J_i is the molar flux diffusive vector (mol/(m^2.s)). The active specific surface area, S_b, is the exposed area of the catalyst pellet to the reacting species (excluding the inside of the pores) in the packed bed and is depicted as:

$$S_b = \frac{3}{r_{pe}}(1 - \varepsilon_b) \quad (8)$$

where r_{pe} is the radius of the catalytic particle (m) and ε_b represents the void fraction of the packed bed.

The molar flux, J_i, is rate determined and is relative to the mass balance and the particle-fluid boundary conditions. This can be displayed based on the external mass transfer coefficient:

$$J_i = h_i(c_i - c_{i,ps}) \quad (9)$$

$$h_i = \frac{Sh \cdot D_i}{2r_{pe}} \quad (10)$$

$$Sc = \frac{\mu}{\rho_M \cdot D_i} \quad (11)$$

$$Re = \frac{2r_{pe} \cdot \rho \cdot u_x}{\mu} \quad (12)$$

$$Sh = 2 + 0.552 Re^{\frac{1}{2}} Sc^{\frac{1}{3}} \quad (13)$$

where h_i is the external mass transfer coefficient (m/s), $c_{i,ps}$ is the species concentration at the surface of the catalyst, and D_i expresses the molecular diffusion coefficient in the bulk (m^2/s). Schmidt (*Sc*), Reynolds (*Re*) and Sherwood (*Sh*) numbers are dimensionless and related to the mass transfer occurring at the pellet-fluid interface. μ and ρ are the viscosity (Pa.s) and density (kg/m^3) of the reacting fluids, respectively.

The bulk diffusivity coefficient, D_i, of the reactant in the fluid phase was estimated using the Reddy-Doraiswamy correlation [5]:

$$D_i = 1 \times 10^{-16} \left(\frac{T\sqrt{M_i}}{\mu V_i^{\frac{2}{3}}} \right) \quad (14)$$

where M_i is the molecular mass of the fluid (g/mol), and V_i is the molar volume at normal boiling point (m^3/kmol).

The heterogeneous chemical reaction that takes place in catalyst pellets can be simulated using the Reactive Pellet Bed feature in COMSOL®. The extra dimension is the radial microscale dimension inside of the catalyst pellet particle. Therefore, the mass balance across a spherical shell at r_{dim} and a predefined 1D extra dimension ($r = r_{dim}/r_{pe}$) is given by:

$$4\pi N\{r^2 r_{pe}^2 \varepsilon_{pe} \frac{\partial c_{pe,i}}{\partial t} + \nabla \cdot \left(-r^2 D_{i,eff} \nabla c_{pe,i}\right) = r^2 r_{pe}^2 R_{pe}\} \quad (15)$$

where N is the number of pellets per unit volume of the bed, $D_{i,eff}$ is the effective diffusion coefficient of the reacting fluid within the pores of the catalyst, and R_{pe} is the reaction rate per unit volume of pellet (mol/(m^3.s)).

The effective diffusion coefficient of the fluids into the pores of the catalytic particle are obtained by relating the diffusion coefficient to either the bulk or the Knudsen diffusivity:

$$D_{i,eff} = \frac{D_i \Phi_p \sigma_c}{\tau} \quad (16)$$

where Φ_p, σ_c and τ are the pellet porosity, constriction factor and tortuosity, respectively. Typical values of these can be found in literature and are, σ_c = 0.8, τ = 3.0 and Φ_p = 0.4 [6].

The Navier-Stokes equation is used for the modelling of the hydrodynamics of the microreactor. The continuity equations demonstrating the conservation of mass and momentum are:

$$\frac{\partial \rho}{\partial t} + \nabla \cdot (\rho u) = 0 \tag{17}$$

$$\rho \frac{\partial u}{\partial t} + \rho (u \cdot \nabla) u = \nabla \cdot [-PI + \tau] + F \tag{18}$$

where P is pressure (Pa), τ is the viscous stress tensor in Pa and F is the volume force vector (N/m^3).

The boundary conditions used for the packed bed reactor in this study are given as:

$$\text{at } x = 0;\ c_i = c_{i,in} \tag{19}$$

$$\text{at } x = x_i;\ \frac{\delta c_i}{\delta x} = 0 \tag{20}$$

$$\text{at y } = 0;\ c_i = 0 \tag{21}$$

$$\text{at } r = 1;\ c_{i,p} = c_{i,ps} \tag{22}$$

$$\text{at } r = 0;\ \frac{\delta c_{i,p}}{\delta r} = 0 \tag{23}$$

3. Results

3.1 Catalyst characterization

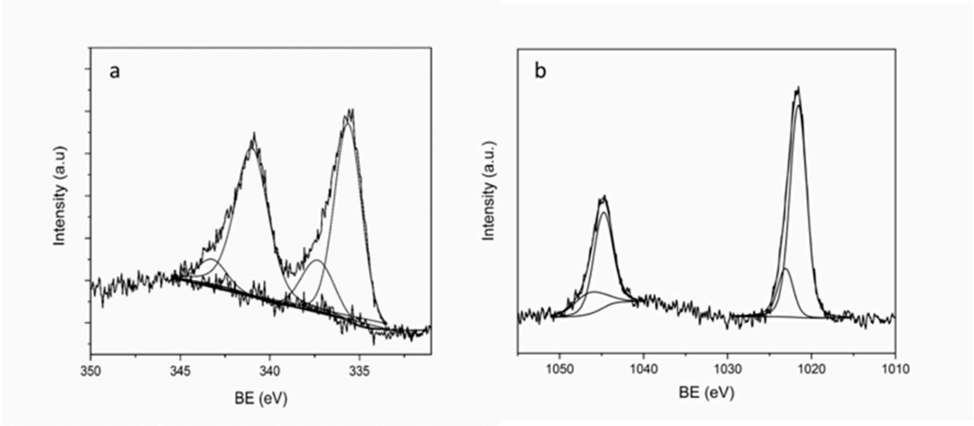

Figure S3. XPS analysis of Pd_6Zn_4 catalyst.

Surface properties of the PdZn catalyst were analysed using XPS analysis , in particular Pd/Zn atomic surface ratio and Pd and Zn oxidation state. The Pd/Zn atomic surface ratio detected by XPS (85-15) is higher than the atomic ratio calculated by STEM-XEDS highlighting an enrichment of Pd on the bimetallic surface compared to the bulk. The binding energies of the Pd 3d3/2 and Pd 3d5/2 electrons were approximately at 341.0 eV and 335.6 eV respectively and could be deconvoluted into two pairs of doublets. The two peaks at high binding energy of 342.8 eV and 337.3 eV were assigned to Pd(II) species, whereas the other two peaks at low binding energy of 340.8 eV and 335.6 eV can be attributed to metallic Pd(0) (Figure S3 a). The percentages of Pd(0) was calculated as 75.8%. Figure S3 b shows the high-resolution XPS spectra for the Zn 2p electrons. The two peaks from the Zn 2p1/2 and Zn 2p3/2 electrons could be deconvoluted into a pair of two peaks, at BE of 1046.1 eV and 1022.7 eV) could be ascribed to Zn(II), whereas the two peaks at 1044.6 eV and 1021.4 eV were assigned to Zn(0) species [7]. The percentages of Pd(0) was calculated as 89.2%.

3.2 Packed bed flow reactor validation

In a previous work conducted by our group [8], different reactor configurations were investigated including single and double membrane packed bed microreactors, using a commercial Pd/C catalyst, to improve the performance of the reaction. The membranes were selectively removing CO from the other gas fluids to avoid the poisoning of the catalyst. The removal of CO prevented and minimised the deactivation of the catalyst prolonging the activity of the catalyst, and also, the separation can enhance the purity of H_2 in order to be used as PEM fuel cell. Figure S4 presents the comparison of the conversion of FA within the single and double membrane configurations at different reactor temperatures.

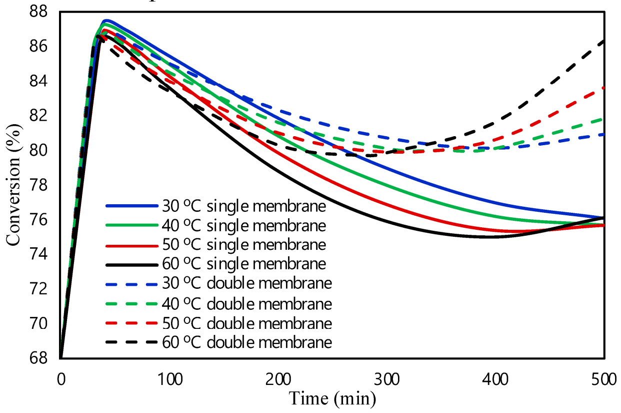

Figure S4. Comparison between the single and double membrane configurations on the formic acid conversion at different temperatures [8].

References

[1] I. Barlocco *et al.*, "Synthesis of palladium-rhodium bimetallic nanoparticles for formic acid dehydrogenation," *Journal of Energy Chemistry*, vol. 52, pp. 301–309, 2021, doi: 10.1016/j.jechem.2020.04.031.

[2] D. A. Bulushev, S. Beloshapkin, and J. R. H. Ross, "Hydrogen from formic acid decomposition over Pd and Au catalysts," *Catalysis Today*, vol. 154, no. 1–2, pp. 7–12, 2010, doi: 10.1016/j.cattod.2010.03.050.

[3] S.-W. Ting, C. Hu, J. K. Pulleri, and K.-Y. Chan, "Heterogeneous Catalytic Generation of Hydrogen from Formic Acid under Pressurized Aqueous Conditions," *Ind. Eng. Chem. Res.*, vol. 51, no. 13, pp. 4861–4867, 2012, doi: 10.1021/ie2030079.

[4] F. Sanchez, D. Motta, A. Roldan, C. Hammond, A. Villa, and N. Dimitratos, "Hydrogen Generation from Additive-Free Formic Acid Decomposition Under Mild Conditions by Pd/C: Experimental and DFT Studies," *Topics in Catalysis*, vol. 61, pp. 254–266, 2018, doi: 10.1007/s11244-018-0894-5.

[5] K. A. Reddy and L. K. Doraiswamy, "Physico-Chemical Constants of Binary Systems in Concentrated Solutions," *Ind. Eng. Chem. Fundamentals*, vol. 6, no. 1, pp. 77–79, 1966.

[6] H. Fogler, "Chapter 10: Catalysis and Catalytic Reactors," in *Elem. Chem. React.*, 5th ed., 2016, pp. 454–456.

[7] Y. Cheng, S. Lu, W. Xu, H. Wen, and J. Wang, "Fabrication of superhydrophobic Au–Zn alloy surface on a zinc substrate for roll-down, self-cleaning and anti-corrosion properties," *Journal of Materials Chemistry A*, vol. 3, no. 32, pp. 16774–16784, Aug. 2015, doi: 10.1039/C5TA03979G.

[8] E. Harkou *et al.*, "Computational Studies on Microreactors for the Decomposition of Formic Acid for Hydrogen Production Using Heterogeneous Catalysts," *Molecules*, vol. 28, no. 14, p. 5399, 2023.
